# Dependence on Mincle and Dectin-2 Varies With Multiple *Candida* Species During Systemic Infection

**DOI:** 10.3389/fmicb.2021.633229

**Published:** 2021-02-25

**Authors:** Aiysha Thompson, Diogo M. da Fonseca, Louise Walker, James S. Griffiths, Philip R. Taylor, Neil A. R. Gow, Selinda J. Orr

**Affiliations:** ^1^Division of Infection and Immunity, Systems Immunity Research Institute, Cardiff University School of Medicine, Cardiff, United Kingdom; ^2^UK Dementia Research Institute, Cardiff, United Kingdom; ^3^School of Medicine, Dentistry and Biomedical Science, Wellcome Wolfson Institute for Experimental Medicine, Queen’s University Belfast, Belfast, United Kingdom; ^4^Aberdeen Fungal Group, University of Aberdeen, Aberdeen, United Kingdom; ^5^Faculty of Dentistry, Oral & Craniofacial Sciences, Centre for Host-Microbiome Interactions, King’s College London, London, United Kingdom; ^6^Medical Research Council Centre for Medical Mycology, University of Exeter, Exeter, United Kingdom

**Keywords:** Mincle, Dectin-2, *Candida*, CLR, fungal

## Abstract

More than 95% of invasive *Candida* infections are caused by four *Candida* spp. (*C. albicans, C. glabrata, C. tropicalis, C. parapsilosis*). C-type lectin-like receptors (CLRs), such as Dectin-1, Dectin-2, and Mincle mediate immune responses to *C. albicans*. Dectin-1 promotes clearance of *C. albicans, C. glabrata, C. tropicalis*, and *C. parapsilosis*, however, dependence on Dectin-1 for specific immune responses varies with the different *Candida* spp. Dectin-2 is important for host immunity to *C. albicans* and *C. glabrata*, and Mincle is important for the immune response to *C. albicans.* However, whether Dectin-2 drives host immunity to *C. tropicalis* or *C. parapsilosis*, and whether Mincle mediates host immunity to *C. glabrata, C. tropicalis* or *C. parapsilosis* is unknown. Therefore, we compared the roles of Dectin-2 and Mincle in response to these four *Candida* spp. We demonstrate that these four *Candida* spp. cell walls have differential mannan contents. Mincle and Dectin-2 play a key role in regulating cytokine production in response to these four *Candida* spp. and Dectin-2 is also important for clearance of all four *Candida* spp. during systemic infection. However, Mincle was only important for clearance of *C. tropicalis* during systemic infection. Our data indicate that multiple *Candida* spp. have different mannan contents, and dependence on the mannan-detecting CLRs, Mincle, and Dectin-2 varies between different *Candida* spp. during systemic infection.

## Introduction

*Candida* spp. are the second most common agents of human fungal infection after dermatophytes (Brown et al., 2012). The vast majority of *Candida* infections (candidiasis) are superficial and pose no serious threat to immunocompetent individuals, however, recurrent mucosal infections impart a significant and widespread morbidity affecting more than 100 million women each year (Kullberg and Arendrup, 2015; Denning et al., 2018). Although invasive candidiasis is much less frequent than other superficial mycoses, it is associated with high mortality rates (Yapar, 2014; Mccarty and Pappas, 2016; Pappas et al., 2018). Indeed, invasive candidiasis is a worrying health concern as it is estimated to affect 250,000 people every year with associated mortality ranging from 46 to 75% (Brown et al., 2012; Bongomin et al., 2017), This high mortality stems mainly from the fact that current antifungal therapies for invasive candidiasis are limited, often administered late in the course of infection, and because the patients are often suffering from one or more predisposing factors (Zaoutis et al., 2005; Lockhart, 2014; Pappas et al., 2018; Sam et al., 2018). Immunotherapy as a potential treatment for invasive candidiasis has yet to be developed, however, this would require an in-depth understanding of the anti-fungal immune response (Segal et al., 2006; van de Veerdonk et al., 2010; Sam et al., 2018).

*C. albicans* is the most virulent and frequently isolated pathogen from patients with invasive candidiasis, accounting for 50–70% of all infections (Pfaller and Diekema, 2007; Guinea, 2014), however, invasive infections caused by other non-*albicans Candida* spp., such as *C. parapsilosis*, *C. glabrata*, and *C. tropicalis* and recently *C. auris* have been rising over the past decades (Arendrup et al., 2002; Yapar, 2014; Lamoth et al., 2018), and pose an emerging health concern. Nevertheless, most research tends to focus on *C. albicans* as a model pathogen of candidiasis, with much less research on other clinically relevant *Candida* spp.

Innate immune cells deploy several molecular mechanisms to control *Candida* proliferation and dissemination into host tissues, and to initiate adaptive immunity in order to ultimately curtail the infection. Innate phagocytes, such as macrophages and dendritic cells (DCs) recognize *Candida* spp. via an array of pattern-recognition receptors (PRRs), such as C-type lectin-like receptors (CLRs) and Toll-like receptors (TLRs) (Patin et al., 2019). These receptors sense pathogen-associated molecular patterns (PAMPs) mainly present in the fungal cell wall, the outermost structure of the *Candida* cell. The CLR, Dectin-1 was long identified as the main fungal β-(1,3)-glucan-binding PRR (Brown et al., 2002; Taylor et al., 2007), and was shown to be involved in the activation of protective immune mechanisms against *C. albicans* and *C. glabrata* via phagocytosis, the respiratory burst, neutrophil extracellular traps release, cytokine/chemokine secretion, recruitment of inflammatory cells and T cell response activation (Taylor et al., 2007; Hardison and Brown, 2012; Branzk et al., 2014; Chen et al., 2017). Dectin-1 has been shown to contribute to host resistance against multiple forms of *C. albicans*-driven candidiasis (Ferwerda et al., 2009; Plantinga et al., 2009, 2010), however, we recently showed that the requirement for Dectin-1 for specific immune responses to *Candida* is species-dependent. We found that Dectin-1 abrogation significantly increases mortality in mice during *C. albicans*-induced systemic candidiasis but does not affect survival following infection with *C. glabrata*, *C. tropicalis* or *C. parapsilosis* (Thompson et al., 2019). However, fungal burden is increased in Dectin-1 KO mice following infection with all four *Candida* spp. (Thompson et al., 2019).

The outer cell wall of *Candida* is dominated by a layer of highly mannosylated cell wall proteins for which the mannan component determines the majority of the molecular mass. Mannans represent a shield that hides the inner glucan layer from recognition by Dectin-1, whilst innate cells recognize a range of mannans in the outer cell wall via a number of CLRs (Gow et al., 2011; Yadav et al., 2020). Mannans can be linked to cell wall proteins via amide linkages (*N*-mannan) or ether linkages [*O*-mannan or via phosphodiester linkages to *N*- or *O*-mannan (phosphomannan)] (Netea et al., 2006; Hall and Gow, 2013). Therefore, host cells rely on various mannan-binding receptors for their recognition including the mannose receptor, DC-SIGN, Galectin-3, Dectin-2, and Mincle. The ligands for these mannan-binding CLRs can either be superficial or buried within the inner cell wall (Vendele et al., 2020). Dectin-2 was shown to bind mannose-rich structures present in the *Candida* cell wall (McGreal et al., 2006; Sato et al., 2006), and to contribute to host protection during systemic infections with *C. albicans* and *C. glabrata* (Ifrim et al., 2014, 2016; Haider et al., 2019; Thompson et al., 2019). In *C. albicans* infections, Dectin-2 was shown to contribute toward the generation of protective Th1 and Th17 responses mainly by promoting production of IL-12p40, IL-1β, and IL-23 by DCs (Robinson et al., 2009; Saijo et al., 2010). Mincle can bind α-mannose residues present in fungal cell wall mannans (Taylor et al., 2005) and also acts as a death sensor by recognizing SAP130, an alarmin released by dying host cells that induces neutrophil infiltration into damaged tissues (Yamasaki et al., 2008). Although it was shown to have a redundant role in *C. albicans* phagocytosis by macrophages, Mincle is recruited to macrophage phagocytic synapse sites, and was shown to be involved in TNF production (Wells et al., 2008). Dependence on Mincle for resistance against *C. albicans*-driven systemic candidiasis appears to be strain-specific, as Mincle was reported to be necessary for controlling *C. albicans* 3630 clearance in the kidneys (Wells et al., 2008), but we recently showed that it plays a redundant role in conferring resistance toward *C. albicans* SC5314 (Thompson et al., 2019). Indeed, other authors have shown that Mincle does not bind multiple *Candida* spp., including *C. albicans* (Yamasaki et al., 2008), making it difficult to ascertain its importance for host protection during candidiasis.

Here we compare the cell wall composition of the four main *Candida* spp. responsible for the vast majority of invasive candidiasis (*C. albicans*, *C. glabrata*, *C. tropicalis*, and *C. parapsilosis*) and determine the role of Mincle and Dectin-2 in response to these different pathogens in a systemic model of infection.

## Materials and Methods

### Mice

C57BL/6J (WT), Mincle KO (*Clec4e*^–/^*^–^*), and Dectin-2 KO (*Clec4n*^–/^*^–^*) mice were maintained and handled according to institutional and U.K. Home Office guidelines. Animals used were age- and gender-matched. In accordance with the 3Rs to reduce mouse numbers, wherever possible the same WT mice were used as controls for multiple KO mice strains in concurrent experiments. Where the same controls have been used for different figures, this is highlighted in the figure legend.

### Ethics Statement

All *in vivo* experimental procedures were approved by and performed in strict accordance to both Cardiff University’s Animal Welfare and Ethical Review Body and the U.K. Home Office. Animal care and use adhered to the Animals (Scientific Procedures) Act 1986.

### Reagents

IFN-γ and IL-17 ELISAs were obtained from R&D. IL-1β, IL-6, IL-10, IL-12p40, and TNF ELISAs were obtained from Life Technologies. M-CSF and GM-CSF used for bone marrow cell differentiation were obtained from Peprotech. Greiss reagent was purchased from Sigma.

### Preparation of *Candida* Cultures

*Candida albicans* SC5314 reference strain was obtained from ATCC. The clinical isolates *Candida glabrata* SCS74761, *Candida tropicalis* SCS74663, *Candida parapsilosis* SCSB588, were kindly donated by Dr. Donna MacCallum (University of Aberdeen). *Candida* spp. were propagated on YPD agar plates overnight at 30°C, then grown in YPD broth for ∼16 h at 30°C in a shaking incubator. *Candida* spp. were washed three times with PBS with centrifugation at 350 × g, for 5 min, and then resuspended at the appropriate concentration for experimental assays.

### *Candida* Preparation for TEM, Cell Wall Thickness, Mannan Fibril Length, and Alcian Blue Assay

High-pressure freezing–freeze substitution transmission electron microscopy (TEM) of *Candida* spp. was performed as previously described (Walker et al., 2018). Briefly, cells were snap-frozen in liquid nitrogen at high pressure using a Leica Empact high-pressure freezer (Leica, Milton Keynes, United Kingdom) and fixed using a Leica AFS freeze substitution system. Samples were then processed in a Lynx tissue processor and embedded in TAAB812 (TAAB Laboratories, Aldermaston, United Kingdom) epoxy resin. One-hundred-nanometer sections were cut with a Leica Ultracut E microtome and stained with uranyl acetate and lead citrate. Samples were imaged with a JEOL 1400 plus transmission microscope (JEOL UK Ltd., Hertfordshire, United Kingdom) and imaging with an AMT UltraVUE camera (Deben, Suffolk, United Kingdom). Five measurements for cell wall thickness and mannan fibril length were taken per cell and 15 cells were measured for each *Candida* spp. Alcian Blue binding assay was used to determine the phosphomannan content of each *Candida* spp., as previously described (Hobson et al., 2004). Briefly, exponentially grown cells from each *Candida* spp. were collected, washed twice with milliQ water and diluted 1 in 10. Cell pellets were resuspended in 1 ml of 30 μg/ml Alcian Blue and incubated at room temperature for 10 min. The cell pellet was then collected and the OD 620_*nm*_ of the supernatant was measured to determine the content of Alcian Blue. The concentration of Alcian Blue bound to cells of each *Candida* spp. was then calculated.

### Cell Culture

Femurs and tibiae of mice were flushed with PBS to harvest bone marrow. Bone marrow cells were cultured for 6 days in DMEM supplemented with 10% heat inactivated fetal bovine serum, 5% heat inactivated horse serum, 2 mM L-glutamine, 100 U/ml penicillin/streptomycin, 10 mM HEPES and 10 ng/ml M-CSF to generate bone marrow-derived macrophages (BMDMs) (Patin et al., 2016). Bone marrow cells were cultured for 8 days in RPMI 1640 medium containing 10% heat inactivated fetal bovine serum, 2 mM L-glutamine, 100 U/ml penicillin/streptomycin, 10 mM HEPES, 1% NEAA, 1 mM sodium pyruvate, 50 μM β-mercaptoethanol and 10 ng/ml GM-CSF to generate bone marrow-derived dendritic cells (BMDCs).

### Cell Stimulations and Cytokine Assays

Differentiated BMDMs were harvested using 8 mg/ml lidocaine, washed and resuspended in RPMI 1640 supplemented with 10% heat inactivated fetal bovine serum and 100 U/ml penicillin/streptomycin. BMDMs/BMDCs were plated in 96-well plates at a density of 1 × 10^5^ cells/well and incubated overnight at 37°C. Media was removed the following day, and cells were stimulated with 1 × 10^5^ *Candida* CFUs/well for 24 h in a final volume of 200 μl fresh media. 2.5 μg/ml amphotericin B was added 2 h after stimulation. Cell culture supernatants were harvested and cytokine levels were measured by ELISA assays, according to the manufacturers’ protocols. Nitric oxide production was detected using the Griess test (Sun et al., 2003). Briefly, following *in vitro* stimulation of host cells with *Candida* spp., cell culture supernatants were harvested and incubated with equal volumes of Griess reagent for 10 min. Absorbance was read at 540_*nm*_ using a Multiskan Spectrum plate reader (Thermo Fisher Scientific) and nitrite levels in samples were calculated from a standard curve generated using a serially diluted sodium nitrite solution.

### *In vivo Candida* spp. Infections

*Candida* spp. was suspended in PBS and intravenously injected in mice in a volume of 100 μl. The *Candida* spp. dose administered varied between different experiments (outlined in figure legends). Mice were weighed daily and examined twice a day by using a predefined scoring system, with 20% body weight loss set as an additional humane endpoint. Mice were culled by CO_2_ asphyxiation and death was confirmed by posterior cervical dislocation. At the end of each experiment blood was collected by cardiac puncture and kidneys, brains and spleens were harvested as previously described (Patin et al., 2016). Serum was extracted from blood by centrifugation at 10,000 rpm for 10 min at 4°C in serum tubes and used in ELISA assays for cytokine detection. The left kidney and right brain were homogenized in 1 mL PBS, homogenates were serially diluted and spotted on petri dishes containing 50 μg/ml chloramphenicol in YPD and incubated for 24–48 h at 30°C. Colonies were counted and fungal burden was calculated as CFUs/g of organ. The spleen was homogenized and erythrocytes were lysed using ACK lysis buffer. Splenocytes were washed by centrifugation and resuspended in IMDM supplemented with 10% heat inactivated fetal bovine serum, 2 mM L-glutamine, 100 U/ml penicillin/streptomycin, 50 μM β-mercaptoethanol. Cells were plated at 1 × 10^6^ cells/well and left unstimulated or stimulated with 2 × 10^6^ CFUs/well *Candida* spp. for 48 h at 37°C. 2.5 μg/ml amphotericin B was added 2 h after stimulation. After 48 h, supernatants were collected and used for IFN-γ and IL-17 ELISA assays.

### Statistical Methods

Data were analyzed using GraphPad Prism. For statistical analysis of two groups, Student’s *t* test was performed, and for three or more groups, One-way ANOVA with Bonferonni’s post-test or Two-way ANOVA with Bonferonni’s post-test was used. Datasets were transformed by Y = sqrt(Y + 0.5) when data did not follow a Gaussian distribution and analyzed by either ANOVA or Student’s *t-*test, or non-parametric tests if normality was not achieved after transformation. One sample *t*-test was used if all the samples were zero in one group. Statistical significance was considered to be achieved when *p*-values were less than 0.05: ^∗^*p* < 0.05, ^∗∗^*p* < 0.01, ^∗∗∗^*p* < 0.001, ^****^p < 0.0001. All data are presented as means ± SEM.

## Results

### *Candida* spp. Display Differences in Their Cell Wall

**FIGURE 1:**
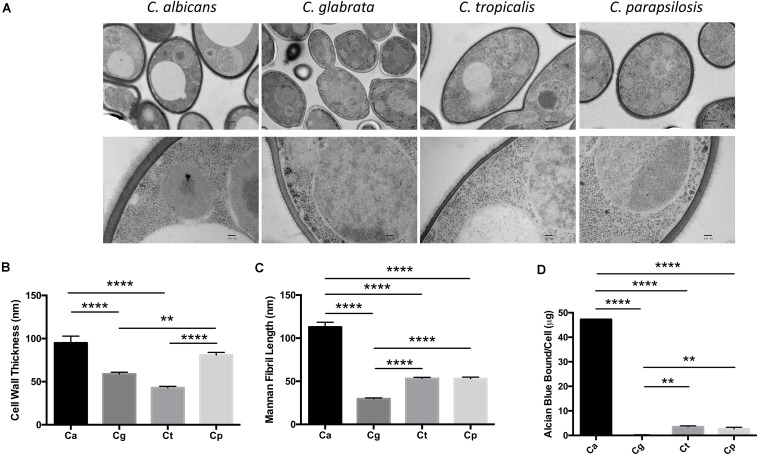
*Candida* spp. yeast display differences in their cell wall. **(A)** Electron micrograph images showing the ultrastructure of *Candida* cells taken at 4,000× (upper panel) and 30,000× (lower panel). Scale bars are 500 nm (upper panel) and 100 nm (lower panel). Images are representative of three separate preparations. **(B,C)** Cell wall thickness **(B)** and mannan fibril length **(C)** were measured from the TEM images. Graphs show the mean ± SEM from 15 *Candida* cells (1-way ANOVA, Bonferroni’s post-test). **(D)** *Candida* spp. were incubated in 30 μg/ml Alcian Blue for 10 min and the level of dye associated with the cell wall was measured by absorbance. Graph displays the mean amount of dye bound per cell ± SEM from three independent experiments (One-way ANOVA with Bonferroni’s post-test). **p < 0.01, ****p < 0.0001.

We compared the cell wall structure of *C. albicans*, *C. glabrata*, *C. tropicalis*, and *C. parapsilosis* by TEM of high-pressure frozen *Candida*. We observed significant differences in cell wall thickness of the inner wall in the following descending order: *C. albicans, C. parapsilosis, C. glabrata, C. tropicalis* (Figures 1A,B). Mannosylated protein fibrils extend out from the inner layer in the TEM images. The *Candida* spp. displayed decreasing mannan fibril length in the following order: *C. albicans, C. parapsilosis = C. tropicalis, C. glabrata* (Figures 1A,C). We then analyzed the cell wall phosphomannan content using Alcian Blue binding. The *Candida* spp. displayed decreasing phosphomannan content in the following order: *C. albicans, C. tropicalis = C. parapsilosis, C. glabrata* (Figure 1D). The significant differences in the *Candida* cell walls particularly in length/type of mannan structures present may affect the immune response mediated by CLRs, such as Dectin-2 and Mincle in response to these different clinically relevant *Candida* spp.

### Mincle and Dectin-2 Regulate Cytokine Responses to Multiple *Candida* spp. in BMDCs

**FIGURE 2:**
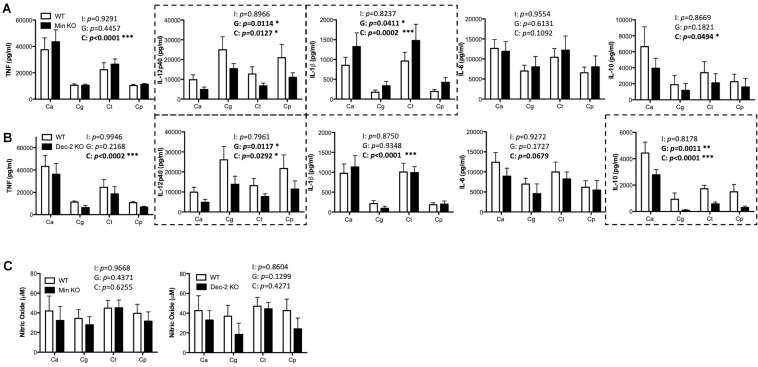
Mincle and Dectin-2 regulate cytokine responses to multiple *Candida* spp. in BMDCs. **(A–C)** BMDCs from WT and Mincle KO mice **(A,C)** or from WT and Dectin-2 KO mice **(B,C)** were stimulated with *C. albicans* SC5314 (Ca), *C. glabrata* SCS74761 (Cg), *C. tropicalis* SCS74663 (Ct), and *C. parapsilosis* SCSB5882 (Cp) at a multiplicity of infection (MOI) of 1:1 (*Candida* sp.: BMDCs). Cytokine levels **(A,B)** and nitric oxide levels **(C)** in the supernatants were measured after 24 h incubation. Results are presented as means ± SEM of 4 **(A)**, 3–4 **(B)**, or 3 **(C)** independent experiments (Two-way ANOVA with Bonferroni’s post-test). I = Interaction between “Genotype” and “*Candida*” variables, G (Genotype) = impact of BMDC genotype (WT vs. Mincle KO; WT vs. Dectin-2 KO), C (*Candida*) = impact of different *Candida* spp. tested (*albicans, glabrata, tropicalis, parapsilosis*). Graphs where Genotype is significant have been surrounded by a dashed box. **(B)** WT data is the same as in **(A)** for three out of three replicates for TNF, three out of four replicates for IL-12p40, IL-1β, and IL-6, and two out of three replicates for IL-10. **(C)** WT data in the right graph is the same as in the left graph for two out of three replicates. *p < 0.05, **p < 0.01, ***p < 0.001.

Mincle has been shown to mediate TNF production from BMDMs in response to *C. albicans* (Wells et al., 2008) while Dectin-2 has been shown to drive cytokine production from BMDCs in response to *C. albicans* mannans (Saijo et al., 2010) and from macrophages during systemic infection with *C. glabrata* (Ifrim et al., 2014, 2016). Here, we investigated the role of Mincle and Dectin-2 during cytokine production from BMDMs and BMDCs in response to four *Candida* spp. To investigate this, BMDMs and BMDCs from WT and Mincle KO or WT and Dectin-2 KO mice were stimulated with *C. albicans* SC5314, *C. glabrata* SCS74761, *C. tropicalis* SCS74663, or *C. parapsilosis* SCSB5882. We did not observe any major roles for Mincle (Supplementary Figures 1A,C) or Dectin-2 (Supplementary Figures 1B,C) in regulating cytokine production or nitric oxide production in response to these four *Candida* spp. This is largely unsurprising as BMDMs do not basally express either Mincle or Dectin-2 (Supplementary Figure 2). However, both Mincle and Dectin-2 regulate cytokine production in response to multiple *Candida* spp. in BMDCs. Mincle mediates IL-12p40 production and inhibits IL-1β production in response to multiple *Candida* spp. (Figure 2A). While Dectin-2 promotes IL-12p40 and IL-10 production in response to multiple *Candida* spp. (Figure 2B). Neither CLR is required for nitric oxide production from BMDCs (Figure 2C). Therefore, Mincle and Dectin-2 regulate cytokine production from BMDCs in response to multiple *Candida* spp. but they are largely uninvolved in cytokine production from BMDMs.

### *In vivo* Clearance of *C. tropicalis* Is Partially Dependent on Mincle

As Mincle regulates cytokine production in response to multiple *Candida* spp. we next sought to determine whether Mincle plays any roles during systemic infection with these different *Candida* spp. To this end, WT and Mincle KO mice were systemically infected with *C. albicans*, *C. glabrata*, *C. tropicalis*, or *C. parapsilosis.*

**FIGURE 3:**
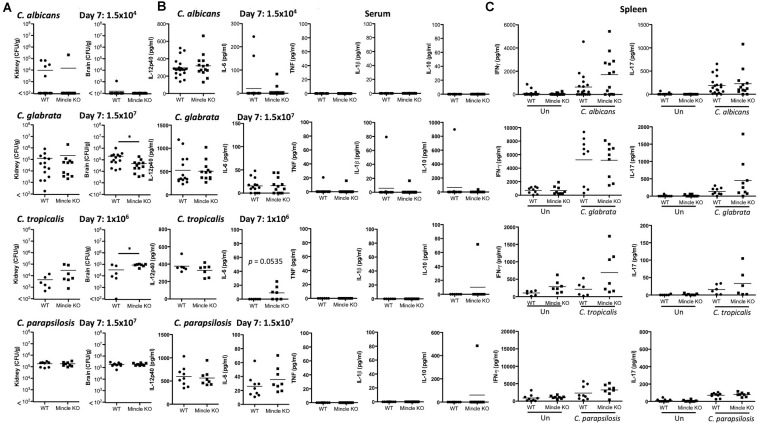
Early clearance of *C. tropicalis* is partially dependent on Mincle. **(A–C)** WT and Mincle KO mice were intravenously infected with the indicated doses of *C. albicans*, *C. glabrata*, *C. tropicalis*, or *C. parapsilosis* for 7 days. **(A)** CFU in the kidneys and brains of mice at 7 days post-infection were determined. Mann-Whitney test (*C. albicans* kidney on transformed data; *C. glabrata* kidney and brain), One sample *t*-test (*C. albicans* brain), Student’s *t-*test (*C. tropicalis* kidney; *C. tropicalis* brain on transformed data; *C. parapsilosis* kidney and brain). **(B)** Cytokine levels in the serum of mice at 7 days post-infection. Student’s *t-*test (*C. albicans* IL-12p40 on transformed data; *C. glabrata* IL-12p40 and IL-6 on transformed data; *C. tropicalis* IL-12p40; C. *parapsilosis* IL-12p40 and IL-6 on transformed data), Mann-Whitney test (*C. albicans* IL-6 on transformed data; *C. glabrata* TNF, IL-1β, and IL-10 on transformed data), One sample *t*-test (*C. tropicalis* IL-6 and IL-10; C. *parapsilosis* IL-10). **(C)** Splenocytes from infected mice were unstimulated or restimulated with live *Candida* spp. for 48 h and IFN-γ and IL-17 levels were measured by ELISA. Kruskal Wallis with Dunn’s post-test (*C. albicans* IFN-γ and IL-17 on transformed data; *C. glabrata* IL-17 on transformed data; *C. parapsilosis* IFN-γ; *C. parapsilosis* IL-17 on transformed data), One-way ANOVA with Bonferroni’s post-test (*C. glabrata* IFN-γ; *C. tropicalis* IFN-γ; *C. tropicalis* IL-17 on transformed data). **(A–C)** Graphs are the cumulative result of 2–4 independent experiments. Each symbol represents an individual mouse. Fifteen out of twenty WT mice in the *C. albicans* graphs, and five out of nine WT mice in the *C. parapsilosis* graphs are the same as Figure 4 in a previous publication from our group (Thompson et al., 2019). *p < 0.05.

**FIGURE 4:**
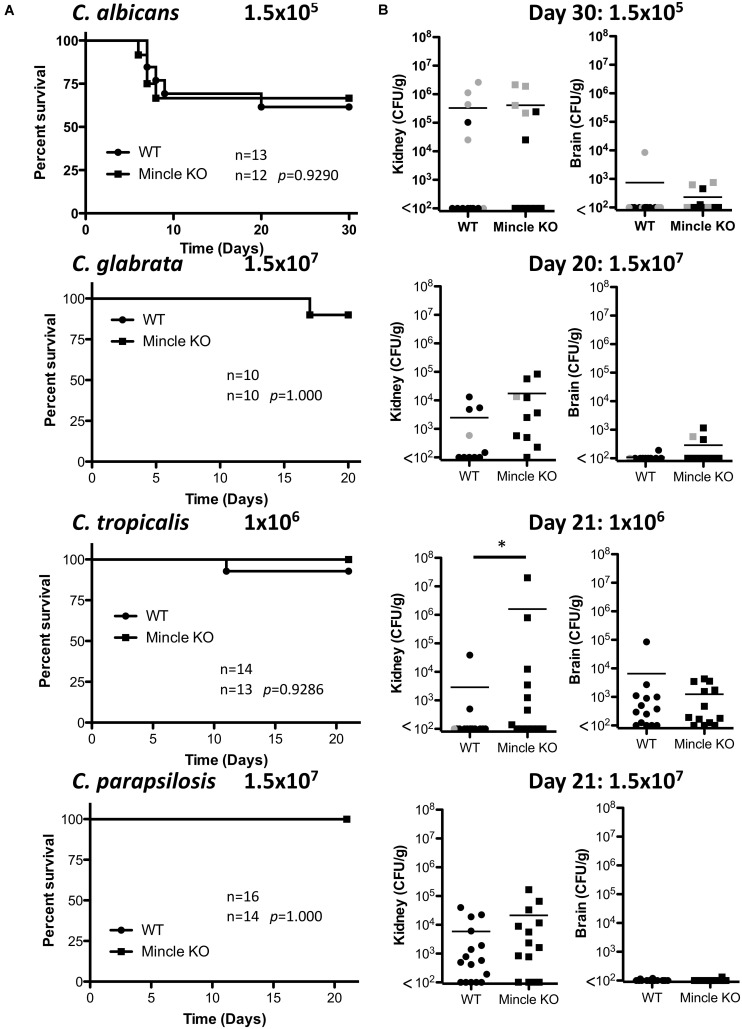
Clearance of *C. tropicalis* is partially dependent on Mincle. **(A)** WT and Mincle KO mice were intravenously infected with the indicated doses of *C. albicans* for 30 days, *C. glabrata* for 20 days, *C. tropicalis* for 21 days, or *C. parapsilosis* for 21 days. Survival curves based on humane end-point of infected WT (filled circles) and Mincle KO mice (filled squares). Graphs are the cumulative result of 2–3 independent experiments. Log-rank test. **(B)** CFU in the kidneys of WT and Mincle KO mice at 20–30 days after infection (black symbols) or at time of death by humane end point (gray symbols). Graphs are the cumulative result of 2–3 independent experiments. Each symbol represents an individual mouse. Mann-Whitney test on transformed data (*C. albicans* kidney and brain; *C. glabrata* brain; *C. tropicalis* kidney and brain; *C. parapsilosis* kidney and brain), Student’s *t-*test on transformed data (*C. glabrata* kidney). Four out of fourteen WT mice in the *C. tropicalis* graphs are the same as Figure 3 of a previous publication from our group (Thompson et al., 2019). *p < 0.05.

The infection doses used were previously determined (Thompson et al., 2019). One-week post-infection, Mincle KO mice showed reduced presence of *C. glabrata* within the brain and decreased clearance of *C. tropicalis* from the brain compared to WT mice (Figure 3A). The WT and Mincle KO mice did not display any other major differences in the early clearance of these four *Candida* spp. from the main target organs. Mincle KO mice also displayed a subtle elevation in serum IL-6 levels 1-week post infection with *C. tropicalis* likely due to the increased fungal burden in these mice (Figure 3B). We did not observe any other major differences in serum cytokine levels between WT and Mincle KO mice following infection with these *Candida* spp. In addition, we tested the ability of WT and Mincle KO splenocytes to produce IFN-γ and IL-17 upon antigen restimulation with live *Candida* spp. in the presence of amphotericin B. We did not observe any major differences in the ability of these splenocytes to produce IFN-γ or IL-17. Finally, we examined the requirement for Mincle for controlling systemic infection with these *Candida* spp. over time. Mincle was largely unimportant for controlling and/or clearing systemic infection with these different *Candida* spp. However, similar to the early clearance of *C. tropicalis*, Mincle once again played a role in the clearance of *C. tropicalis* over time as the kidneys in Mincle KO mice displayed increased fungal burden compared to WT mice after 21 days (Figures 4A,B). Mincle is therefore relatively unimportant for controlling systemic *Candida* infections with the specific strains used in this study, however, it plays a role during systemic infection with *C. tropicalis*.

### Dectin-2 Is Important for *in vivo* Clearance of Multiple *Candida* spp.

**FIGURE 5:**
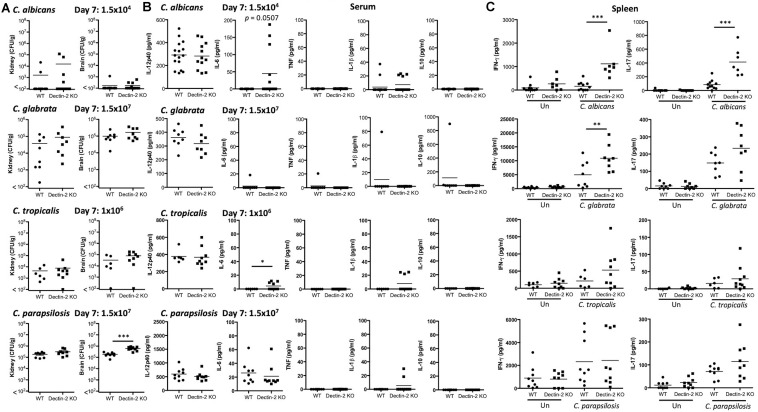
Early clearance of *C. parapsilosis* is partially dependent on Dectin-2. **(A–C)** WT and Dectin-2 KO mice were infected intravenously with the indicated doses of *C. albicans*, *C. glabrata*, *C. tropicalis*, or *C. parapsilosis* for 7 days. **(A)** CFU in the kidneys and brains of mice at 7 days post-infection were determined. Mann-Whitney test (*C. albicans* kidney and brain on transformed data), Student’s *t-*test (*C. glabrata* kidney and brain on transformed data; *C. tropicalis* kidney and brain on transformed data; *C. parapsilosis* kidney and brain). **(B)** Cytokine levels in the serum of mice at 7 days post-infection. Student’s *t-*test (*C. albicans* IL-12p40; *C. albicans* IL-1β on transformed data; *C. glabrata* IL-12p40; *C. tropicalis* IL-12p40), Mann-Whitney test (C. *parapsilosis* IL-12p40 and IL-6), One sample *t*-test (*C. albicans* IL-6; *C. glabrata* IL-6, TNF, IL-1β, and IL-10; *C. tropicalis* IL-6 and IL-1β; C. *parapsilosis* IL-1β). **(C)** Splenocytes from infected mice were unstimulated or restimulated with live *Candida* spp. for 48 h and IFN-γ and IL-17 levels were measured by ELISA. One-way ANOVA with Bonferroni’s post-test (*C. albicans* IFN-γ and IL-17 on transformed data; *C. glabrata* IFN-γ; *C. glabrata* IL-17 on transformed data; *C. tropicalis* IFN-γ and IL-17 on transformed data; *C. parapsilosis* IFN-γ and IL-17 on transformed data). **(A–C)** Graphs are the cumulative result of 2–4 independent experiments. Each symbol represents an individual mouse. Seven out of sixteen WT mice in the *C. albicans* graphs **(A,B)** and 7 out of 11 WT mice in the *C. albicans* graphs **(C)** are the same as Figure 3. Four out of eight WT mice in the *C. glabrata* graphs are the same as Figure 4 in a previous publication from our group (Thompson et al., 2019). Four out of eight WT mice in the *C. glabrata* graphs, six out of six WT mice in the *C. tropicalis* graphs, and nine out of nine WT mice in the *C. parapsilosis* graphs are the same as Figure 3 in a previous publication from our group (Thompson et al., 2019). *p < 0.05, **p < 0.01, ***p < 0.001.

**FIGURE 6:**
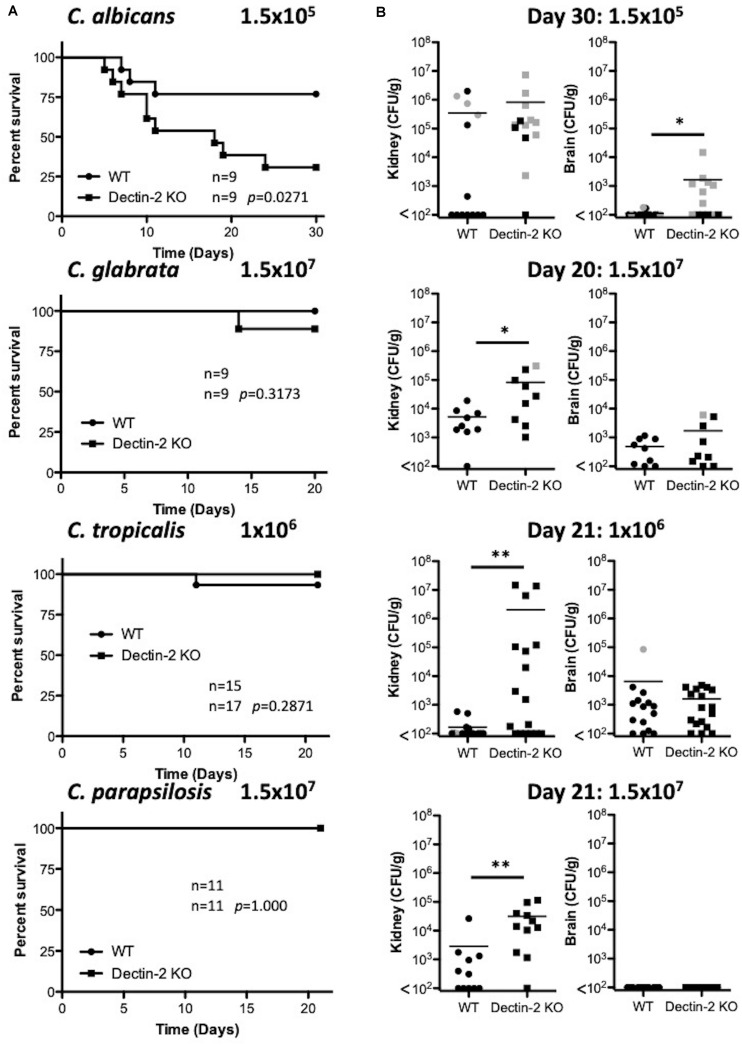
Dectin-2 is important for clearance of multiple *Candida* spp. **(A)** WT and Dectin-2 KO mice were infected intravenously with the indicated doses of *C. albicans* for 30 days, *C. glabrata* for 20 days, *C. tropicalis* for 21 days, or *C. parapsilosis* for 21 days. Survival curves based on humane end-point of infected WT (filled circles) and Dectin-2 KO mice (filled squares). Graphs are the cumulative result of 2–3 independent experiments. Log-rank test. **(B)** CFU in the kidneys of WT and Mincle KO mice at 20–30 days after infection (black symbols) or at time of death by humane end point (gray symbols). Graphs are the cumulative result of 2–3 independent experiments. Each symbol represents an individual mouse. Mann-Whitney test on transformed data (*C. albicans* kidney and brain; *C. tropicalis* kidney and brain; *C. parapsilosis* kidney), Student’s *t-*test on transformed data (*C. glabrata* kidney and brain), One sample *t*-test (*C. parapsilosis* brain). Nine out of nine WT mice in the *C. glabrata* graphs are the same as Figure 3 and 10 out of 14 WT mice in the *C. tropicalis* graphs are the same as Figure 4 of a previous publication from our group (Thompson et al., 2019). *p < 0.05, **p < 0.01.

As Dectin-2 mediates cytokine production in response to multiple *Candida* spp. and it has been shown to play a role during infections with *C. albicans* (Saijo et al., 2010) and *C. glabrata* (Ifrim et al., 2014, 2016), we next analyzed the role for Dectin-2 at different timepoints during systemic infection with these four clinically relevant *Candida* spp. To this end, WT and Dectin-2 KO mice were systemically infected with *C. albicans*, *C. glabrata*, *C. tropicalis*, or *C. parapsilosis*. One-week post-infection, Dectin-2 KO mice showed decreased clearance of *C. parapsilosis* from the brain compared to WT mice (Figure 5A). The WT and Dectin-2 KO mice did not display any other major differences in the early clearance of these four *Candida* spp. from the main target organs. We did not observe any major differences in serum cytokine levels between WT and Dectin-2 KO mice following infection with these *Candida* spp., however, Dectin-2 KO mice displayed a subtle elevation in serum IL-6 levels 1-week post infection with *C. albicans* or *C. tropicalis* (Figure 5B). Dectin-2 KO splenocytes produced more IFN-γ upon antigen restimulation with live *C. albicans* and *C. glabrata* and increased IL-17 production following restimulation with *C. albicans* (Figure 5C). Finally, we examined the requirement for Dectin-2 for controlling systemic infection with these *Candida* spp. over time. Dectin-2 was important for clearance of all four *Candida* spp. (Figures 6A,B). Thus, Dectin-2 is important during early infection with *C. parapsilosis* and at later timepoints for controlling systemic *Candida* infections with all four *Candida* spp.

## Discussion

In this study, we demonstrated that the cell wall thickness and mannan composition varied between *C. albicans* and three other clinically relevant, non-*albicans* species. We found that Mincle and Dectin-2 are involved in regulating cytokine production in response to all four *Candida* spp. However, in an *in vivo* model of systemic infection, we showed that Mincle is involved in clearance of *C. tropicalis* while Dectin-2 is involved in the clearance of all four *Candida* spp. Overall, we found that both Mincle and Dectin-2 play roles in response to different *Candida* spp. however this is host cell-, species- and likely strain- and site of infection-dependent.

As Mincle and Dectin-2 bind mannose/mannans in fungal cell walls, we hypothesized that differences in the cell wall mannan content between these four clinically relevant *Candida* spp. would determine whether Mincle or Dectin-2 are involved in the immune response to the different *Candida* spp. Mincle has been shown to bind mannose (Lee et al., 2011) and Dectin-2 has been shown to bind high mannose structures (e.g., Man_9_GlcNAc_2_) (McGreal et al., 2006). Therefore, we compared the cell wall mannan content of these four clinically relevant *Candida* spp. Interestingly, we found large differences in the mannan fibril length and phosphomannan content. This would indicate significant differences in the N-glycan structures on the surface of these four *Candida* spp. In agreement with our data, Nguyen et al. (2018) found that the molecular weights of *Candida* mannans decreased in the following order: *C. albicans, C. tropicalis, C. glabrata*. The predicted structures of these mannans differed substantially with much larger and more complex *C. albicans* mannans compared to the other *Candida* spp. (Nguyen et al., 2018). In addition, Shibata et al. (2007) showed that the mannan structure changed as *C. albicans* yeast transformed to hyphae. Moreover, several factors like different morphogenetic and morphological stages of *Candida* spp., the physicochemical properties of the substrate, such as the carbon source, pH, oxygen levels, metal ion micronutrients and temperature, and also certain environmental stresses, such as the presence of different antifungal drugs were shown to alter carbohydrate synthesis and orientation in the fungal cell wall (Ballou et al., 2016; Pradhan et al., 2019; Vendele et al., 2020). Therefore, various factors will likely determine whether specific PRRs, such as Mincle and Dectin-2 are important for immune responses to these different *Candida* spp. These include: differences in the mannan content of the four *Candida* spp. when grown under the same conditions; the complexity of the mannans on the fungal cell wall at yeast, and likely hyphal stages; and the fact that some of these species do not form hyphae.

In this study, we next assessed whether Mincle or Dectin-2 influenced cytokine release and nitric oxide production in response to these four *Candida* spp. In BMDCs, we found that Mincle mediates IL-12p40 production and regulates IL-1β production, while Dectin-2 drives production of IL-12p40 and IL-10 upon *Candida* spp. stimulation. Wells et al. (2008) reported that Mincle was involved in TNF production from macrophages following stimulation with *C. albicans* 3630. We did not observe any defect in TNF production in Mincle KO BMDMs or BMDCs. The difference between our results and those previously published by Wells et al. (2008) could be due to the timepoints (1–2 h vs. 24 h) or the strains of *C. albicans* (3630 vs. SC5314) used in the experiments. Dectin-2 was previously shown to be important for production of IL-12p40, TNF, IL-1β, and IL-10 from BMDCs in response to *C. albicans* mannans (Saijo et al., 2010). In addition, naïve Dectin-2 KO peritoneal macrophages generally produced lower levels of TNF, IL-6, KC, IL-1α, and IL-1β compared to WT controls in response to *C. albicans* (Ifrim et al., 2016). However, cytokine production from naïve Dectin-2 KO peritoneal macrophages was similar to WT cells in response to *C. glabrata* (Ifrim et al., 2014). The different results could be due to the use of different cell types and whole *C. albicans* vs. *C. albicans* mannans. The altered IL-12p40 and IL-1β levels that we observed in Mincle KO BMDCs and the attenuated IL-12p40 and IL-10 levels in Dectin-2 KO BMDCs would suggest that despite significant differences in the mannan levels/structures on the different *Candida* spp. that ligands for both these CLRs are present on *C. albicans*, *C. glabrata*, *C. tropicalis*, and *C. parapsilosis.* While we observed roles for Mincle and Dectin-2 in mediating/regulating cytokine production from BMDCs, this may differ for myeloid cell populations in target organs, such as the kidney or spleen.

As Mincle regulated cytokine production *in vitro* in response to these four *Candida* spp., we then assessed whether Mincle had any impact during systemic infection. We did not observe any major effect of Mincle deficiency on the clearance of *C. albicans* or *C. parapsilosis* either at early or late timepoints. This is in agreement with our previous findings (Thompson et al., 2019), while Wells et al. (2008) observed increased fungal burden in Mincle KO mice 5 days after systemic infection with *C. albicans*. The *C. albicans* strain, time point and dose differed between our studies and that by Wells et al. (2008). In our study, Mincle KO mice displayed slightly enhanced early clearance of *C. glabrata* from the brain, however, fungal burden was similar to WT at later timepoints and cytokine production was unaltered suggesting a minimal role. Interestingly, we found that although Mincle does not significantly influence survival, it plays a role in the clearance of *C. tropicalis* from the brain and kidneys of systemically infected mice at early and late timepoints. In previous studies with *C. albicans*, we have observed elevated IL-6 levels in the serum of mice with higher fungal burdens, and our data with Mincle KO mice suggest a similar trend during infection with *C. tropicalis* (Orr et al., 2013; Thompson et al., 2019). While we identified a role for Mincle in regulating cytokine production from all four *Candida* spp. *in vitro*, we only observed a major role for Mincle in regulating the clearance of *C. tropicalis in vivo*. Based on our findings we cannot rule out other roles for Mincle *in vivo* in response to these four *Candida* spp., however, our data indicate that while a ligand for Mincle may be present on all four *Candida* spp. tested, Mincle is only important for the clearance of *C. tropicalis* during systemic infection.

Similar to our experiments with Mincle, we then assessed whether Dectin-2 had any impact during systemic infection with all four *Candida* spp. We found that Dectin-2 is important for clearance of all *Candida* spp. tested, and that it significantly contributes to survival during *C. albicans* infection. The *C. albicans* and *C. glabrata* data is in agreement with previous findings (Saijo et al., 2010; Ifrim et al., 2014, 2016; Thompson et al., 2019). Serum IL-6 was also elevated to a significant or near significant level in Dectin-2 KO mice following infection with *C. albicans* and *C. tropicalis* similar to our previous observations (Orr et al., 2013; Thompson et al., 2019). Splenocytes from *C. albicans*- or *C. glabrata*-infected Dectin-2 KO mice displayed enhanced IL-17 and/or IFN-γ production following restimulation with the same *Candida* sp. Ifrim et al. (2016) also showed similar enhanced T-cell associated cytokine production from Dectin-2 KO splenocytes following infection with *C. albicans* and restimulation with heat-killed *C. albicans*, although the time points differed from our study. However, in contrast to our findings, Ifrim et al. (2014) showed reduced T-cell associated cytokine production following infection with *C. glabrata* and restimulation with heat-killed *C. glabrata.* In addition, IL-17 production by splenocytes following infection with *C. albicans* was decreased with the addition of anti-Dectin-2 (Robinson et al., 2009). The experimental setup for each of these findings differ considerably, which could potentially explain some of the discrepancies. However, we believe that the enhanced IL-17 and/or IFN-γ production from the Dectin-2 KO splenocytes in our study is likely associated with enhanced T cell exposure to the *Candida* spp. *in vivo* due to the inability of these mice to clear/control these infections. Saijo et al. (2010) showed reduced T-cell associated cytokine production from purified naïve Dectin-2 KO CD4^+^ T cells following stimulation with supernatants from BMDCs cultured with *C. albicans* indicating an inability of Dectin-2 KO T cells to appropriately respond to *C. albicans*. Therefore, our data indicate that Dectin-2 is important for the clearance of all four *Candida* spp., similar to our *in vitro* cytokine findings, however, roles for Dectin-2 in specific responses *in vivo* during systemic infection varies with *Candida* spp. Our data does not rule out additional roles for Dectin-2 in mediating production of other cytokines in response to these four *Candida* species or at different sites of infection.

At the start of this study, we postulated that differences in host immune responses and consequent *in vivo* fungalclearance of four clinically relevant *Candida* spp. could be due to different mannan levels/structures on the cell wall. While we found significant differences in the mannan fibril length and phosphomannan in the four *Candida* spp., our results do not suggest a consistent relationship between type/amount of mannan and Mincle or Dectin-2 involvement in cytokine production or fungal clearance. Spatial orientation and exposure of different mannan ligands on the *Candida* cell wall was recently shown to greatly influence recognition by mannan-binding CLRs, including Dectin-2 (Vendele et al., 2020). It is possible that differences in cell wall architecture of the distinct *Candida* spp. tested here, could translate to different exposure of these ligands and greatly influence the ability of immune cells to recognize them via Mincle and Dectin-2. An in-depth analysis of the cell wall and the mannan structure on these *Candida* spp. may help to identify a pattern that could connect a specific mannan structure/type that is associated with each CLR, however, from our data, this pattern is not currently evident. Overall in this study, we have shown that Dectin-2 in particular, but also Mincle are important for *Candida*-mediated immune responses during systemic infection, although the level of CLR importance varies with species and likely strain of *Candida*.

## Data Availability Statement

The raw data supporting the conclusions of this article will be made available by the authors, without undue reservation.

## Ethics Statement

The animal study was reviewed and approved by the Cardiff University’s Animal Welfare and Ethical Review Body.

## Author Contributions

AT designed and performed the experiments. DF performed the experiments and wrote the manuscript. LW and JG performed the experiments. PT and NG guided the research. SO conceptualized and guided the research, designed and performed the experiments, and wrote the manuscript. All authors contributed to the manuscript revision, and read and approved the submitted version.

## Conflict of Interest

The handling editor declared a shared affiliation with one of the authors JG at time of review. The authors declare that the research was conducted in the absence of any commercial or financial relationships that could be construed as a potential conflict of interest.

## Supplementary Material

Supplementary Figure 1 *Candida*-induced responses in BMDMs are largely independent of Mincle and Dectin-2. **(A–C)** BMDMs from WT and Mincle KO mice **(A,C)** or from WT and Dectin-2 KO mice **(B,C)** were stimulated with *C. albicans* SC5314 (Ca), *C. glabrata* SCS74761 (Cg), *C. tropicalis* SCS74663 (Ct), and *C. parapsilosis* SCSB5882 (Cp) at a MOI of 1:1 (*Candida* sp.: BMDMs). Cytokine levels **(A,B)** and nitric oxide levels **(C)** in the supernatants were measured after 24 h incubation. Results are presented as means ± SEM of 4–7 **(A)**, 3 **(B)**, or 3 **(C)** independent experiments (Two-way ANOVA with Bonferroni’s post-test). I = Interaction between “Genotype” and “*Candida*” variables, G (Genotype) = impact of BMDM genotype, C (*Candida*) = impact of different *Candida* spp. tested. **(A)** WT data is the same as in^26^, for 2 out of 4–7 replicates for TNF, IL-12p40, IL-1β, IL-6, and IL-10. **p* < 0.05, ***p* < 0.01, ****p* < 0.001.

Supplementary Figure 2 CLR Expression on BMDMs and BMDCs. Representative flow cytometry histograms showing basal Mincle and Dectin-2 surface expression and Mincle expression following 4 h LPS stimulation on BMDMs and BMDCs. Plots are representative of 2–3 independent experiments. Dashed line = isotype control, solid line = CLR.
